# Dimensional Gradient Structure of CoSe_2_@CNTs–MXene Anode Assisted by Ether for High-Capacity, Stable Sodium Storage

**DOI:** 10.1007/s40820-020-00562-7

**Published:** 2021-01-04

**Authors:** Enze Xu, Pengcheng Li, Junjie Quan, Hanwen Zhu, Li Wang, Yajing Chang, Zhenjie Sun, Lei Chen, Dabin Yu, Yang Jiang

**Affiliations:** 1 School of Materials Science and Engineering, Hefei University of Technology, Hefei, 230009 People’s Republic of China; 2 School of Chemistry and Chemical Engineering, Hefei University of Technology, Hefei, 230009 People’s Republic of China; 3 State Key Laboratory of Pulsed Power Laser Technology, National University of Defense Technology, Hefei, 230037 People’s Republic of China

**Keywords:** CoSe_2_@CNTs–MXene, Ether electrolyte, In situ XRD, DFT calculation, Sodium-ion full battery

## Abstract

Recently, abundant resources, low-cost sodium-ion batteries are deemed to the new-generation battery in the field of large-scale energy storage. Nevertheless, poor active reaction dynamics, dissolution of intermediates and electrolyte matching problems are significant challenges that need to be solved. Herein, dimensional gradient structure of sheet–tube–dots is constructed with CoSe_2_@CNTs–MXene. Gradient structure is conducive to fast migration of electrons and ions with the association of ether electrolyte. For half-cell, CoSe_2_@CNTs–MXene exhibits high initial coulomb efficiency (81.7%) and excellent cycling performance (400 mAh g^−1^ cycling for 200 times in 2 A g^−1^). Phase transformation pathway from crystalline CoSe_2_–Na_2_Se with Co and then amorphous CoSe_2_ in the discharge/charge process is also explored by in situ X-ray diffraction. Density functional theory study discloses the CoSe_2_@CNTs–MXene in ether electrolyte system which contributes to stable sodium storage performance owing to the strong adsorption force from hierarchical structure and weak interaction between electrolyte and electrode interface. For full cell, CoSe_2_@CNTs–MXene//Na_3_V_2_ (PO_4_)_3_/C full battery can also afford a competitively reversible capacity of 280 mAh g^−1^ over 50 cycles. Concisely, profiting from dimensional gradient structure and matched electrolyte of CoSe_2_@CNTs–MXene hold great application potential for stable sodium storage.

**Electronic supplementary material:**

The online version of this article (10.1007/s40820-020-00562-7) contains supplementary material, which is available to authorized users.

## Highlights

- Dimensional gradient structure of sheet–tube–dots was constructed with CoSe_2_@CNTs–MXene for fast ion and electron transportation.
- Density functional theory study discloses the electrochemical difference of CoSe_2_@CNTs–MXene in ether/ester electrolyte system.
- Phase transformation of CoSe_2_@CNTs–MXene was analyzed by in situ XRD. The full cell based on CoSe_2_@CNTs–MXene anode was also assembled.

**Electronic supplementary material:**

The online version of this article (10.1007/s40820-020-00562-7) contains supplementary material, which is available to authorized users.

## Graphical abstract

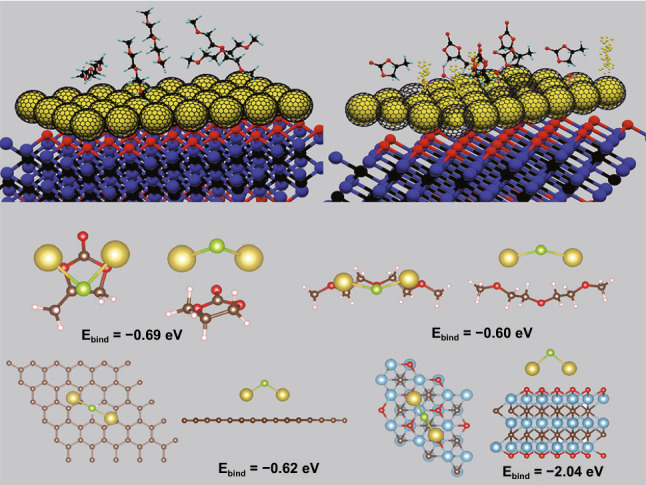

## Introduction

Recently, emerging sodium-ion batteries (SIBs) have been deemed to be the new-generation battery for large-scale energy storage applications benefitting for abundant resources, low cost and low standard hydrogen potential (− 2.71 V) [1–4]. In the last ten years, researchers have developed a great variety of anode electrode materials and different kinds of cathode materials for SIBs [5, 6]. Meanwhile, advanced SIBs systems, such as Na–S, Na–metal and Na–O_2_ batteries, have also achieved prominent energy density in the effort of researchers [7–9]. However, owing to its large ionic radius (1.02 Å) compared to Li ion (0.76 Å) and dense molar mass, poor reaction kinetics, bad rate performance and weak cycling performance limit the large-scale application of SIBs [10]. Therefore, constructing high specific capacity anode materials with good reaction kinetics is an effective method to build extraordinary energy density sodium-ion full cells. Meanwhile, appropriate electrolyte systems are another way to ensure long-term and high cycling performance for full batteries. So, it is necessary to develop compatible electrode materials and electrolyte system to promote the application of SIBs.

Transition metal chalcogenides (TMCs), a kind of narrow/zero bandgap materials with low cost, high electric conductivity and unique electrochemical properties, have been widely investigated as the potential active materials for alkali-ion batteries [11–14]. In most cases, pulverization caused by huge volume change and dissolution of intermediate in electrochemical processes leads to poor cycling performance. A generic strategy is encapsulating TMCs in functionalized carbon transferred from metal–organic frameworks (MOFs) [15]. MOFs can provide high surface area and controllable microstructure. After carbonization and selenylation process, the hollow functionalized carbon shell can tolerate tremendous phase change stress [16]. Meanwhile, heterogeneous element-doped carbon shell can also restrain the dissolution of sodium selenide in electrolyte [17]. Xu et al. synthesized ZnSe/N-doped hollow carbon architectures for SIBs with a revisable capacity of 250.8 mAh g^−1^ at 1 A g^−1^ exhibiting excellent stability [18]. CoSe_2_@N-CF/CNTs can give a capacity of 428 mAh g^−1^ at 1 A g^−1^ after cycling for 500 times [19]. Synergistic effect in hierarchical gradient structure can greatly enhance electron/ion diffusion, as well as carbon shell encapsulation strategy can guarantee the stabilization of active materials and impede the strain during electrochemical processes. Therefore, constructing hierarchical structure of TMCs transferring from MOFs is a potential method to acquire high-performance sodium-ion anode materials.

Electrolyte, an ion conductor, is another critical component of the rechargeable battery system [20]. Common carbonate ester-based solvents such as ethylene carbonate (EC) and propylene carbonate (PC) are widely applied in SIBs. These electrolyte systems can obtain high ionic conductivity, wide electrochemical window and stable solid electrolyte interphase (SEI) film on the surface of electrode materials [21]. However, low initial coulombic efficiency (ICE), poor cycling stability and the dissolution of reaction intermediate are severe challenges that need to be addressed. Compared with LIBs, the working voltage is usually lower than 4 V, which makes it possible for the adoption of ester electrolyte system [22]. On the other hand, the ester-based electrolyte can significantly improve ICE and rate performance of SIBs [23, 24]. Transition metal sulfides (TMDC) like ZnS showed a superior rate capability and outstanding long-term cyclability assisted by ether electrolyte [25]. Cu_2_MoS_4_–RGO exhibited excellent cycling stability (215 mAh g^−1^ after 2000 cycles) and good full cell performance (75.5% after 500 cycles) [26].

Herein, we report a dimensional gradient structure building with CoSe_2_@CNTs–MXene anode materials for SIBs by exploiting a NaPF_6_ in a new DEGDME electrolyte system. Cobalt (Co)–MOFs are deposited on the MXene by an easy coprecipitation method. Carbon nanotubes (CNTs) grow on the surface of MXene in the catalysis of Co particles, followed by selenylation process. MXene, acting as the flexible matrix, not just promotes the fast ion and electronic transmission by constructing a “sheet–tube–dots” hierarchical structure, but also impedes the dissolution of Na_2_Se in electrochemical processes. The CoSe_2_@CNTs–MXene in ether electrolyte maintains an outstanding cycling performance of 400 mAh g^−1^ after 200 cycles at 2 A g^−1^ with a high ICE of 81.7% and excellent rate stability of 347.5 mAh g^−1^ at 5 A g^−1^, which is much better than electrochemical behaviors in ester system (only 27 mAh g^−1^ for 200 cycles). The great electrochemical contrast of CoSe_2_@CNTs–MXene in disparate electrolyte systems is evidenced by DFT calculations. Meanwhile, phase transformation of CoSe_2_@CNTs–MXene in the first cycle was successfully analyzed by in situ XRD and dynamic electrochemical impedance spectroscopy (EIS) analysis. Importantly, CoSe_2_@CNTs–MXene and Na_3_V_2_ (PO_4_)_3_/C full cell is assembled, delivering outstanding cycle performance with a capacity of 280 mAh g^−1^ after 50 cycles at 100 mA g^−1^.

## Experimental Section

### Materials

2-Methylimidazole, Co (NO)_3_ 6H_2_O, methanol, LiF and HCl were purchased from Shanghai Aladdin Bio-Chem Technology Co., Ltd. Ti_3_AlC_2_ powders were purchased from Jilin 11 Technology Co. Ltd., China. All chemical reagents were used without further purification. The ultrapure water was used throughout the experiment process.

### Synthesis of Ti_3_C_2_T_x_ MXene Nanosheets

Firstly, 1 g LiF was dissolved in 10 mL 9 M HCl at room temperature. Then, 1 g Ti_3_AlC_2_ powders were added into LiF/HCl solution for 10 min to avoid overheating. The next, mixed solution was transferred into a Teflon autoclave and kept at 60 ℃ for 24 h. After cooling to room temperature, the etching product was washed with 3 M HCl and ultrapure water successively, until the pH of solution reached 7. Finally, the black jelly was sonicated in water under the protection of argon for 2 h and centrifuged at 3500 rpm. The concentration of final obtained Ti_3_C_2_T_x_ MXene nanosheets colloidal solution was about 8 mg mL^−1^.

### Synthesis of CoSe_2_@CNTs–MXene

In a popular method, 0.6 mmol Co (NO)_3_·6H_2_O and 10 mL MXene colloidal solution were mixed in 40 mL methanol. Then, 6 mmol 2-methylimidazole in 50 mL methanol with 12 μL triethylamine was poured into the above solution followed by stirring continuously for 1 h. After washing with methanol, the ZIF-67@MXene was dried in vacuum at 80 ℃ for 12 h. Next, ZIF-67@MXene was annealed under Ar/H_2_ atmosphere at 700 ℃ for 2 h with a heating rate for 2 ℃ min^−1^, the Co@CNTs–MXene. At last, Co@CNTs–MXene was mixed with Se powder at a weight ratio of 1:3 and heated under Ar atmosphere at 500 ℃ for 3 h. The preparation of CoSe_2_@CNTs was synthesized by a similar method without MXene.

### Materials Characterization

X-ray diffraction (XRD) was tested by Rigaku SmartLab SE with Cu Kα radiation (λ = 1.5406 Å). Thermogravimetric analysis (TGA) was measured by STA 449 F5 Jupiter. Raman spectrum was obtained by LabRAM HR Evolution using 532-nm laser. The surface composition of samples was analyzed by X-ray photoelectron spectroscopy (XPS, ESCALAB250Xi). Morphology was acquired by a field emission scanning electron microscope (FESEM, ZEISS SIGMA) and a field emission transmission electron microscope (FETEM, JEOL JEM-2100F); both of them were equipped with an EDX spectrometer (Oxford Instruments). In situ XRD was represented by a special electrochemical reaction unit with a beryllium (Be) window.

### Electrochemical Measurements

For half-cell, active materials (AC), Super P and PVDF were stirred with a weight ratio of 8:1:1 by a high-speed homogenizer. The slurry was coated on copper foil with a scraper, and the mass load of AC is about 1.1 mg cm^−2^. Sodium foil and glass fiber (Whatman) were used as a counter electrode and a separator. Two different kinds of system ether (1 M NaPF_6_ in DEGDME) and ester (1 M NaPF_6_ in PC) were adopted as electrolyte, respectively. And the ratio of electrolyte/electrode in the half-cell is about 10 uL mg^−1^. For full cell, Na_3_V_2_ (PO_4_)_3_/C was adopted as cathode materials. The capacity matching was achieved by controlling the coating thickness and promoting the anode capacity having a 5–10% surplus compared with cathode capacity. The mass loading of cathode electrode slice is about 6 mg cm^−2^. Before assembling full cell, CoSe_2_@CNTs–MXene was activated for three cycles in advance. Cyclic voltammetry (CV) curves were obtained by CHI 660D workstation. A Neware BTS-4008 system was employed for charge/discharge and rate performance. Electrochemical impedance spectroscopy (EIS) was evaluated by using a Zahner IM6 system (0.01–10^5^ Hz).

### Computational Details

Quantum ESPRESSO v6.4.1 software packages were adopted for all density functional theory (DFT) calculation [27]. Perdew–Burke–Ernzerhof (PBE) in generalized gradient approximation (GGA) considering van der Waals force (VDW) was employed to analyze the exchange functional [28]. The kinetic energy cutoffs for the wavefunction were 60 Ry. 5 × 5 × 1 C–N surface and 3 × 3 × 1 Ti_3_C_2_O_2_ surface built with a 20 Å vacuum region. A 3 × 3 × 1 k-point mesh of Brillouin zone was put to use. The binding energy (*E*_bind_) of Na_2_Se on different matrix could be calculated by *E*_bind_ = *E*_Na2Se-matrix_-*E*_Na2Se_-*E*_matrix_. For all structure and adsorption models, the energy convergence accuracy was within 1 × 10^–7^ eV and 0.001 eV Å for force. Rietveld method was used to refine the XRD data of Na_3_V_2_ (PO_4_)_3_/C [29].

## Results and Discussion

### Synthesis and Characterization of CoSe_2_@CNTs–MXene

**Fig. 1:**
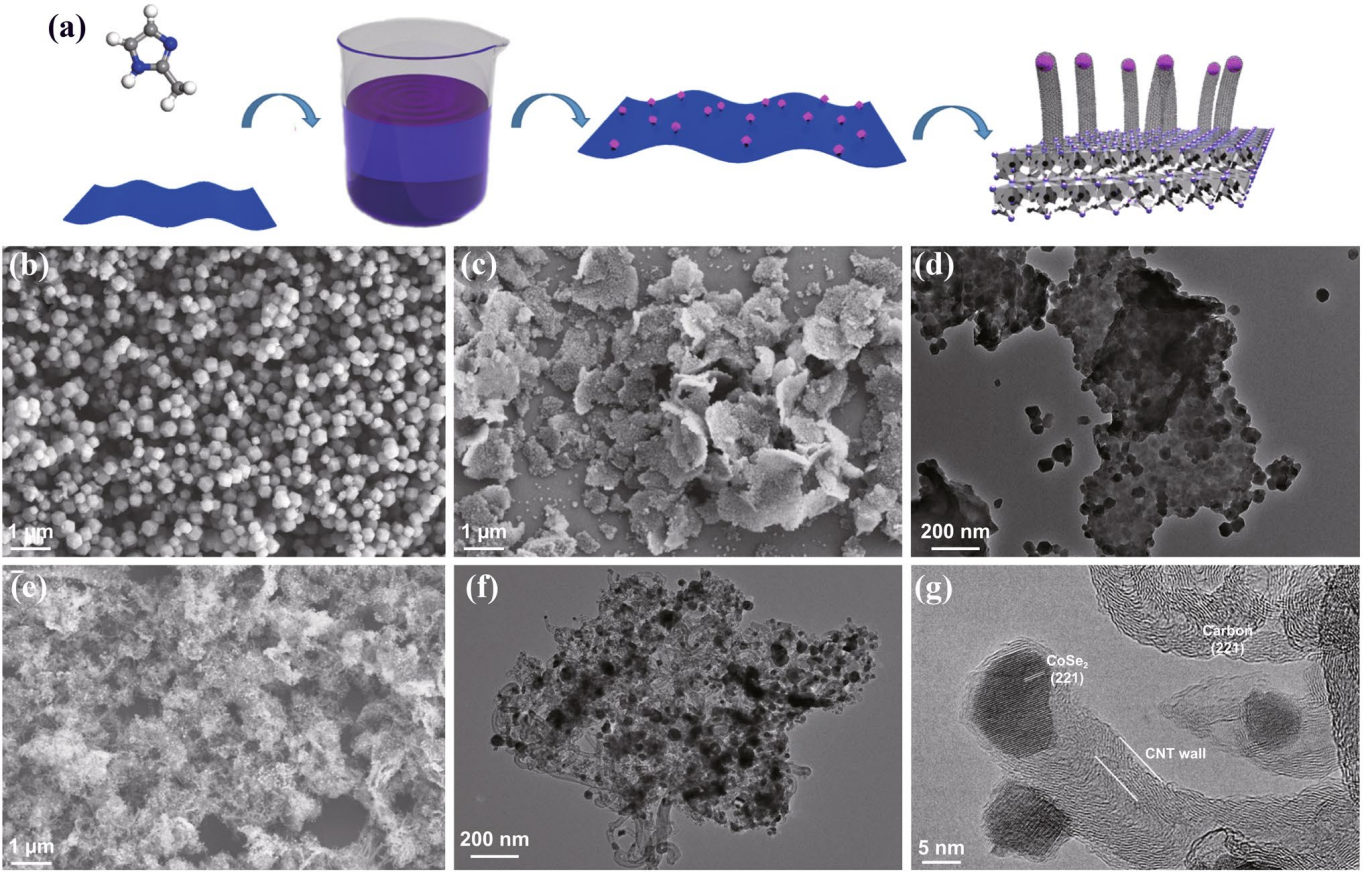
**a** Synthesis scheme of CoSe_2_@CNTs–MXene. **b** SEM image of ZIF-67. **c** SEM image of ZIF-67/MXene. **d** TEM image of ZIF-67/MXene. **e** SEM image of Co/CNTs-MXene. **f** TEM image of CoSe_2_@CNTs–MXene. **g** HRTEM image of CoSe_2_@CNTs–MXene

CoSe_2_@CNTs–MXene was prepared by a universal strategy followed by carbonization and selenylation processes, which is displayed in Fig. 1a. Typically, single-layer Ti_3_C_2_T_x_ was synthesized through a fluoride-based salt etchants method reported by Ghidiu [30, 31]. To avoid the oxidization of Ti_3_C_2_T_x_ during usage, appropriate sodium *L*-ascorbate was added in the colloidal solution [32]. XRD patterns (Fig. S1) indicate successful synthesis of Ti_3_C_2_T_x_ MXene. Microstructure of single-layer Ti_3_C_2_T_x_ characterized by TEM is shown in Fig. S2, and the individual Ti_3_C_2_T_x_ MXene looks ultrathin and transparent, with a size range from 100 to 700 nm [33]. Selected area electron diffraction (SAED) patterns reveal excellent crystallinity and unique hexagonal structure of single-layer Ti_3_C_2_T_x_ MXene [34]. Then, ZIF-67 nanocubes were prepared through a conventional co-precipitation method at ambient temperature [35]. SEM images of ZIF-67 (Fig. 1b) exhibit homogeneous octahedrons with a diameter of 150–250 nm. For ZIF-67/MXene, ZIF-67 with an average size of 25 nm covered the entire surface of MXene (Fig. 1c, d) evenly under the effect of electrostatic interaction [36]. The growth of ZIF-67 could well impede the reunion of MXene benefitting for construction the conductive network. XRD analysis in Fig. S3 also clearly shows the similarity between ZIF-67 and ZIF-67/MXene. After annealing treatment at 800 ℃ under Ar/H_2_ atmosphere, a novel “tube-on-nanosheet” structure of Co@CNTs–MXene was obtained (Fig. 1e). CNTs with hundreds of nanometers grew neatly on the surface of MXene. Co^2+^ ions were reduced to elemental cobalt (ICPDS No.15–0806) under the effect of H_2_ (Fig. S4). Pure cobalt nanoparticles constantly catalyze the growth of carbon nanotubes during the carbonization process of ZIF-67 [37]. The final product CoSe_2_@CNTs–MXene was acquired after selenylation at 500 ℃ for 3 h, as displayed in Fig. 1f, g. CoSe_2_ particles were encapsulated on the top of CNTs. Two crystal interplanar spacings, 0.253 and 0.319 nm, could be well fitted with the (221) of CoSe_2_ and (103) of carbon, respectively. The elemental mapping of CoSe_2_@CNTs–MXene is also displayed in Fig. S5. For comparison, CoSe_2_@CNTs was also prepared without the addition of MXene (Fig. S6). The construction of a “sheet–tube–dots” hierarchical structure can significantly enhance the transport of electrons and sodium ions.

**Fig. 2:**
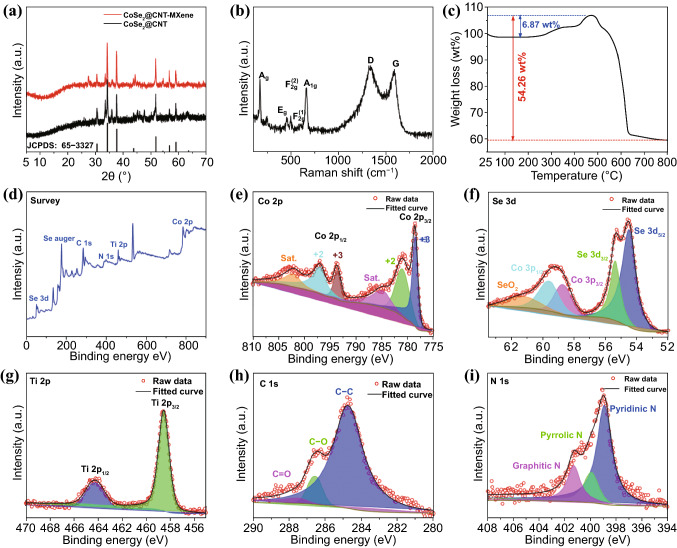
**a** XRD patterns of CoSe_2_@CNTs–MXene and CoSe_2_@CNTs. **b** Raman spectrum of CoSe_2_@CNTs–MXene. **c** TG analysis of CoSe_2_@CNTs–MXene. **d** XPS survey spectrum. **e–i** Co 2p, Se 3d, Ti 2p, C 1 s and N 1 s of CoSe_2_@CNTs–MXene

The XRD patterns of CoSe_2_@CNTs and CoSe_2_@CNTs–MXene are displayed in Fig. 2a. Both of them clearly show a single-phase composition of cubic CoSe_2_ (ICPDS no. 65–3327, space group: Pa $\stackrel{\mathrm{-}}{3}$). No additional reflection appearing in the spectrogram means the high purity of products. At the same time, characteristic peaks of MXene at 5–10° could not be found in the diagram. This phenomenon indicates MXene has been evenly dispersed in CoSe_2_@CNTs–MXene. Raman spectroscopy (Fig. 2b) of CoSe_2_@CNTs–MXene shows two peaks at 186 and 673 cm^−1^, respectively, owning to the A_g_ and A_1g_ modes for CoSe_2_. Some tiny peaks near 500 cm^−1^ can be attributable to the slight oxidation of CoSe_2_ surface. *D* and *G* peaks for *sp*^3^ and *sp*^2^ carbon are situated at 1345 and 1586 cm^−1^, respectively. The *I*_D_/*I*_G_ scale is about 1.07, indicating a good degree of graphitization, which is conducive to the transfer of electrons and sodium ions.

TGA of CoSe_2_@CNTs–MXene was tested in hot air from 25 to 800 ℃ with a heating rate of 10 ℃ min^−1^, as shown in Fig. 2c. The mass increases between 400 and 500 ℃ owing to the formation of SeO_2_ and oxidization of MXene, respectively. The primary mass loss after 600 ℃ contributes to the transformation from CoSe_2_ to Co_3_O_4_ and the sublimation of SeO_2_ [19]. Based on the above TGA tests, the calculated mass percentage of CoSe_2_ in CoSe_2_@CNTs–MXene is about 63%. XPS was measured to invesitage chemical states of CoSe_2_@CNTs–MXene, total spectrogram of CoSe_2_@CNTs–MXene (Fig. 2d) is clearly observed the coexistence of Co, Se, Ti, C and N, relatively.  Figure 2e–i exhibits the fine spectrogram of Co 2p, Se 3d, Ti 2p, C 1 s and N 1 s, respectively. The signal of Co 2p (Fig. 2e) shows two broad peaks locating at 781.1 and 797.2 eV corresponding to Co 2p_3/2_ and Co 2p_1/2_. Peaks at 778.4 and 793.7 eV can be corresponded to the Co–O bond due to the surface oxidization of CoSe_2_ [38]. Owing to the orbital between Co atoms and Se atoms, two satellite peaks also can be found in the spectrogram [35]. The Se 3d spectra (Fig. 2f) can be split into two main peaks at 54.5 and 55.4 eV for Se 3d_5/2_ and Se 3d_3/2_, respectively. Peaks between 57 and 62 eV belong to the existence of CoSe_2_ and SeO_2_ [39]. In the high-resolution Ti 2p spectra (Fig. 2g), peaks at 458.55 and 464.3 eV correspond to Ti 2p_3/2_ and Ti 2p_1/2_ [40]. For C 1 s in Fig. 2h, three peaks locating at 284.8, 286.6 and 287.55 eV can be assigned to *sp*^2^ C, C–O and C = O, respectively. Peaks from left to right for N 1 s are attributed to graphitic, pyrrolic and pyridinic nitrogen, in turn [18].

### Electrochemical Performance of CoSe_2_@CNTs–MXene

**Fig. 3:**
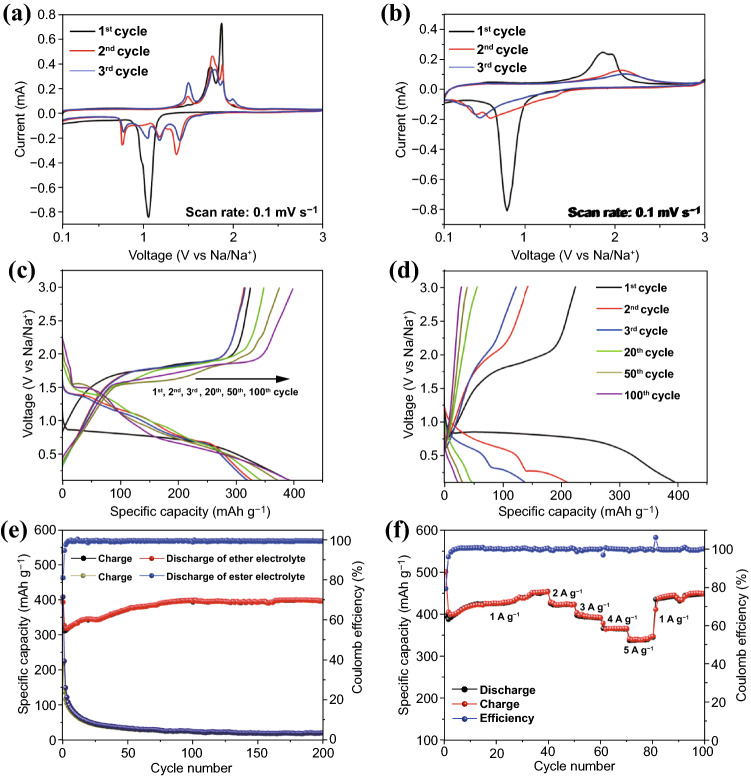
**a, b** CV curves of CoSe_2_@CNTs–MXene in ether/ester electrolyte at a scan rate of 0.1 mV s^−1^ in the range of 0.1–3 V. **c, d** Discharge/charge curves of CoSe_2_@CNTs–MXene in ether/ester electrolyte. **e** Cycle performance of CoSe_2_@CNTs–MXene in ether and ester electrolyte at a current of 2 A g^−1^. **f** Rate performance of CoSe_2_@CNTs–MXene in ether

**Fig. 4:**
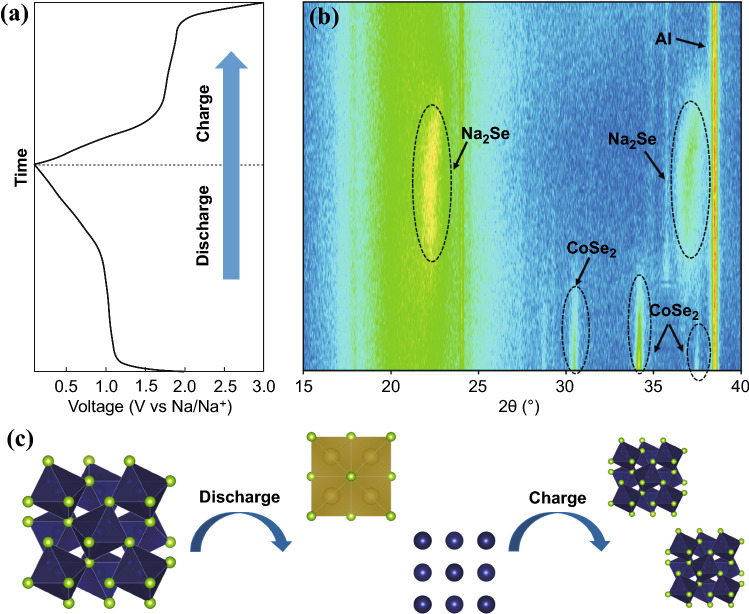
**a** Discharge/charge curves of CoSe_2_ in the first cycle at 100 mA g^−1^. **b** Corresponding in situ XRD patterns of CoSe_2_ in the first cycle. **c** Transformation mechanism of CoSe_2_

The unique structure of CoSe_2_@CNTs–MXene makes it a promising application in energy storage fields. Electrochemical performances of CoSe_2_@CNTs–MXene were investigated by assembling half-cell countering with sodium foil. Cyclic voltammetry (CV) curves of CoSe_2_@CNTs–MXene (Fig. 3a) in ether electrolyte were measured in 0.1–3 V at a scan rate of 0.1 mV s^−1^. In the first discharge, a broad cathodic peak at 1.065 V corresponds to the insertion of sodium ion into CoSe_2_, leading to the formation of Na_x_CoSe_2_. With further discharge, Na_x_CoSe_2_ was eventually broken into Na_2_Se and Co till to 0.1 V. Then, in the anodic process, peaks locating at 1.7–2.0 V stand for Na_2_Se, which regenerated to amorphous CoSe_2_ clusters [38, 41, 42]. For comparison, CV curves of CoSe_2_@CNTs are also displayed in Fig. S7. In order to reveal the electrochemical reaction progress of the first cycle, in situ XRD was also tested, which is displayed in Fig. 4. When the voltage decreased from 2.0 to 1.0 V, diffraction peaks of CoSe_2_ at 30.5°, 34.2° and 37.6° gradually vanished. At this stage, sodium ion embedded into CoSe_2_ and further formed Na_x_CoSe_2_. With the voltage down to 0.1 V, Na_x_CoSe_2_ was decomposed completely and peaks of Na_2_Se became apparent. In the charging process, Na_2_Se has sloughed the sodium ion and transformed to CoSe_2_ again. Reaction processes can be described as Eqs. 1 and 2:

1 $${\text{CoSe}}_{{2}} + {\text{ xNa}}^{ + } + {\text{ e}}^{ - } \leftrightarrow {\text{Na}}_{{\text{x}}} {\text{CoSe}}_{{2}}$$

2 $${\text{Na}}_{{\text{x}}} {\text{CoSe}}_{{2}} + \, \left( {{4} - {\text{x}}} \right){\text{ Na}}^{ + } \leftrightarrow {\text{Co }} + {\text{ Na}}_{{2}} {\text{Se}}.$$

Due to the highly disordered structure of CoSe_2_ in charge course, the characteristic peaks of CoSe_2_ cannot be discovered in the spectra. In order to illustrate this mechanism clearly, ex situ Raman was measured. After charging to 3 V, the characteristic peak of CoSe_2_ is found in Fig. S8. All these pieces of evidence can prove the CoSe_2_–Na_2_Se–CoSe_2_ conversion process.

Figure 3b exhibits the CV graphs of CoSe_2_@CNTs–MXene in the ester environment, which presents a distinct electrochemical process. The cathodic peak in 0.8 V in the first cycle shifts left about 0.26 V than ether electrolyte, and a loose anodic peak exists around 2 V. However, reaction peaks could not fit commendably and disappeared gradually after the second cycle. Galvanostatic charge/discharge profiles of CoSe_2_@CNTs–MXene in ether at a current of 2 A g^−1^ are shown in Fig. 3c. In the first cycle, the discharge/charge capacities can be evaluated to 397.5/324.6 mAh g^−1^, with high coulombic efficiency (CE) about 81.7%. Irreversible capacity loss in the first cycle can be attributed to the generation of solid electrolyte interface (SEI) film. At the same time, the plateaus in discharge/charge processes can exhibit well fitness with CV curves. Reversible capacity for CoSe_2_@CNTs–MXene in ether retains at 315.7, 317.2, 346.6, 376.8 and 397.7 mAh g^−1^ after 2nd, 3rd, 20th, 50th and 100th cycles, with a CE of about 99.8%. Continuous capacity increase benefits from the improvement in electrode wettability and capacitance effect in electrochemical processes. On the other hand, a great deal of capacity loss was observed at CoSe_2_@CNTs–MXene in ester; discharge/charge capacities in the first cycle are 413/223.9 mAh g^−1^ with a low CE of 54.2% (Fig. 3d). Side reactions of electrolyte and serious dissolution of selenide lead to the low coulombic efficiency in the first cycle. Reversible capacity after 2nd, 3rd, 20th, 50th and 100th cycles is only 143.5, 122.9, 56.8, 40 and 31 mAh g^−1^, respectively. With the cycle proceeding in progress, discharge/charge plateaus disappear gradually and only matrix materials contributive capacity exists after 200 cycles. Cycle performances of CoSe_2_@CNTs–MXene in the different electrolyte systems at 2 A g^−1^ are displayed in Fig. 3e. CoSe_2_@CNTs–MXene in the ether can keep up a stable capacity at 400 mAh g^−1^ after 200 cycles, which is vastly superior to a low capacity of 27 mAh g^−1^ for the electrode in ester. For CoSe_2_@CNTs and pure MXene, the revisable capacity is only 215.34 mAh g^−1^ after 200 cycles (Figs. S9 and S10), respectively. Rate performances of CoSe_2_@CNTs–MXene in the ether (Fig. 3f) can obtain invertible capacities for 450.5, 423, 391.5, 366 and 347.5 mAh g^−1^ at a current of 1, 2, 3, 4 and 5 A g^−1^, respectively, which is superior to some other MXene-based anodes (Table S1). All of these performance tests indicate CoSe_2_@CNTs–MXene can exhibit great electrochemical properties in ether electrolyte. In order to explain the huge electrochemical performance discrepancy of CoSe_2_@CNTs–MXene in different electrolyte systems, XPS investigation for C 1 s, O 1 s and F 1 s of anode materials (after the first cycle) is shown in Fig. S11. The SEI layer comprises sodium inorganic/organic complex, organic matter and fluoride. For ether, an obvious peak locating at 686.7 eV can be contributed to the C–F bond. Such high fluorine content guarantees prominent mechanical strength of SEI layer, which can keep the cycling stability during electrochemical processes [7]. After cycling for 100 times, separators were disassembled for XPS analysis. Spectra in Fig. S12 can also verify the dissolution of selenide. A weak peak ranging from 58 to 60 eV can be found in the ester spectra, which belongs to selenide signals. In contrast, no signal was detected in the ether separator.

### DFT Calculations

**Fig. 5:**
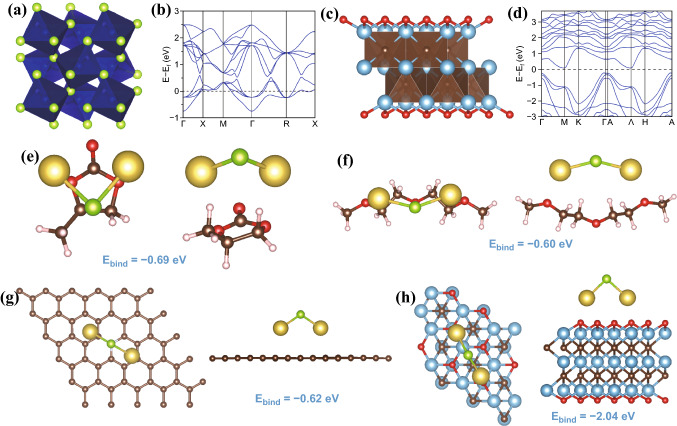
**a–d** Crystal structure and band structure of CoSe_2_ and Ti_3_C_2_O_2_. **e–h** Optimized adsorption structure of PC/Na_2_Se, DEGDME/Na_2_Se, C-N/Na_2_Se and Ti_3_C_2_O_2_/Na_2_Se

Density functional theory (DFT) was used to describe the polyselenide shuttling constraint mechanism in different electrolytes. The space group of cubic CoSe_2_ (Fig. 5a) is Pa $\stackrel{\mathrm{-}}{3}$, which can be well fitted with XRD data. Band structure of CoSe_2_ demonstrates its metallic behavior and outstanding electron conductivity. For MXene, the bandgap of Ti_3_C_2_O_2_ is only 0.3 eV. In our previous research, sodium ion can migrate easily on the surface of MXene. CoSe_2_ nanoparticles, carbon nanotubes and MXene nanosheets together construct a spot–line–surface system that contributes to the ultrafast kinetics for ion transport and electron conduction. The lowest unoccupied molecular orbital (LUMO) and highest occupied molecular orbital (HOMO) of propylene carbonate (PC) and bis (2-methoxy ethyl) ether (DEGDME) are shown in Fig. S13. Energy gaps of PC and DEGDME are 6.0363 and 5.2606 eV, respectively; both of them can satisfy the requirement of SIBs. Relaxed adsorption geometries of Na_2_Se/PC, Na_2_Se/DEGDME, Na_2_Se/C-N and Na_2_Se/ Ti_3_C_2_O_2_ are demonstrated in Fig. 5e–h. The binding energy between PC, DEGDME and Na_2_Se is 0.69 and 0.6 eV. It follows that PC as the electrolyte solvent has an interaction with sodium selenide. DFT calculation further confirmed the binding strength of Na_2_Se on C–N nanosheet is only 0.62 eV. So, when Na_x_CoSe_2_ transferred into Na_2_Se and pure Co in the first discharge process, abundant Na_2_Se dissolved in the electrolyte, leading to the low initial coulombic efficiency and serious capacity loss. Charge density difference of Na_2_Se on Ti_3_C_2_O_2_ (Fig. S14) shows an electron-loss region around selenium atoms and an electron-rich region around MXene surface. Exposed Ti atoms and O atoms on MXene could capture electrons from selenium atoms under the effect of the Lewis acidity property [43]. Ti–Se bond and Se–O bond guarantee Na_2_Se can be anchored tightly (2.04 eV) on the surface of MXene [42]. The enormous binding energy prevents the dissolution of Na_2_Se in the process of electrochemical processes, which is in good agreement with experimental results.

### Kinetics Analysis of CoSe_2_@CNTs–MXene

**Fig. 6:**
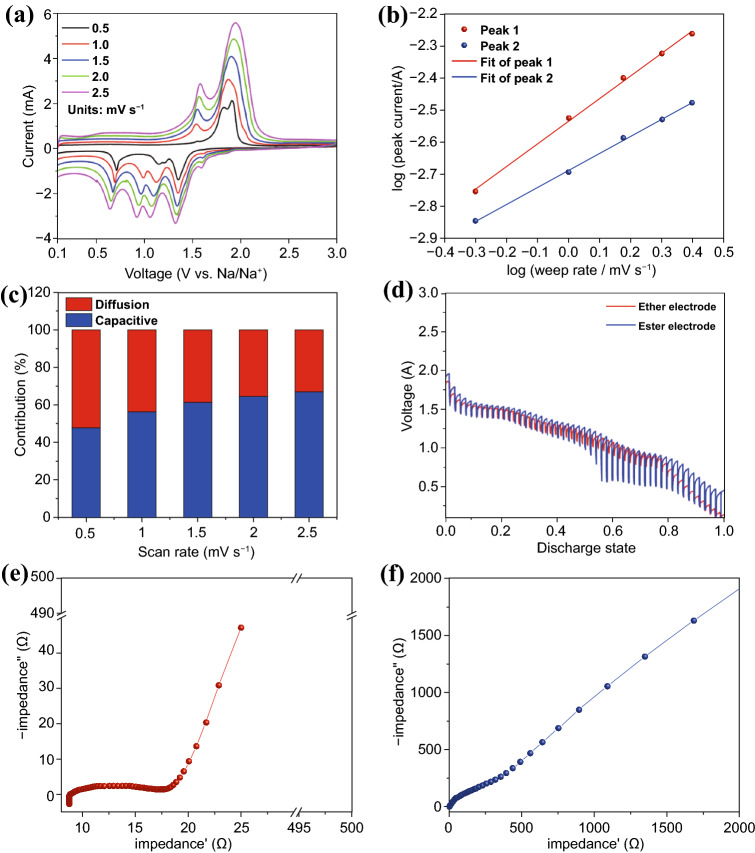
**a** CV curves of CoSe_2_@CNTs–MXene at various currents from 0.5–2.5 mV s^−1^. **b** log *i* versus log *v* plots. **c** Contribution ratio of capacitive capacity of CoSe_2_@CNTs–MXene at various scan rates. **d** GITT of CoSe_2_@CNTs–MXene in ether at a current of 100 mA g^−1^. **e, f** Nyquist plots of CoSe_2_@CNTs–MXene in ether/ester electrolyte

Capacitive/diffusion behavior analysis, galvanostatic intermittent titration technique (GITT) and electrochemical impedance spectroscopy (EIS) are adopted to investigate the excellent electrochemical kinetics of CoSe_2_@CNTs–MXene in detail. The cathode and anode peaks of CoSe_2_@CNTs–MXene (Fig. 6a) at different scan rates (from 0.5 to 2.5 mV s^−1^) show no significant deviation and can be repeatable at a high scan rate. This evidence can also explain the reason for outstanding rate performances of CoSe_2_@CNTs–MXene. The plot log (i) against log (v) and the fitting line are displayed in Fig. 5b. Calculation formulas of the capacitive contribution are Eqs. S1 and S2 described detail in supporting information. Capacitive contribution for specific capacity is 47.6, 56.2, 61.2, 64.5 and 67%, respectively, under the scan rate of 0.5, 1.0, 1.5, 2.0 and 2.5 mV s^−1^ (Fig. 6c). Higher contribution of capacitance at high scan rate is conducive to favorable cycle and rate properties [44, 45]. Diffusion stability of sodium ion in CoSe_2_@CNTs–MXene under different electrolytes could get further insight by GITT. Figure 6d shows the discharge curve from 3.0 to 0.1 V under an identical current of 100 mA g^−1^ for 5 min followed by a relaxation step for 30 min until the cutoff voltage. The ether battery shows evident cycling stability than ester than the good reaction kinetics of CoSe_2_@CNTs–MXene in ether electrolyte. Electrochemical impedance spectroscopy analysis of CoSe_2_@CNTs–MXene in different electrolytes was also explored. Nyquist plots of CoSe_2_@CNTs–MXene in ether and ester are displayed in Fig. 6e–f. EIS of CoSe_2_@CNTs was also tested (Fig. S15). EIS curves can be divided into three parts, inductive reactance and semicircle related to the resistance of electrode in high-frequency region and the linear part for ion diffusion in low frequency [46]. Both curves can be well fitted with the classic equivalent circuit model as shown in Fig. S16. Charge-transfer resistance between interfaces for CoSe_2_@CNTs–MXene in ether is 9.77 Ω, which is much lower than the ester system (239 Ω). Other parameters are also listed in Table S2. This phenomenon also proves the favorable invasion at interface. Low resistance for the interface of electrode and electrolyte contributes to high sodium-ion diffusion rate during electrochemical processes. Dynamic EIS analysis of CoSe_2_@CNTs–MXene in different kinds of electrolyte at first discharge/charge cycle (Fig. S17) can also prove the excellent reaction kinetics of ether. The addition of MXene can also improve interface situations owing to the contact of the solid–liquid interface.

### Full Cells Evaluation of CoSe_2_@CNTs–MXene

**Fig. 7:**
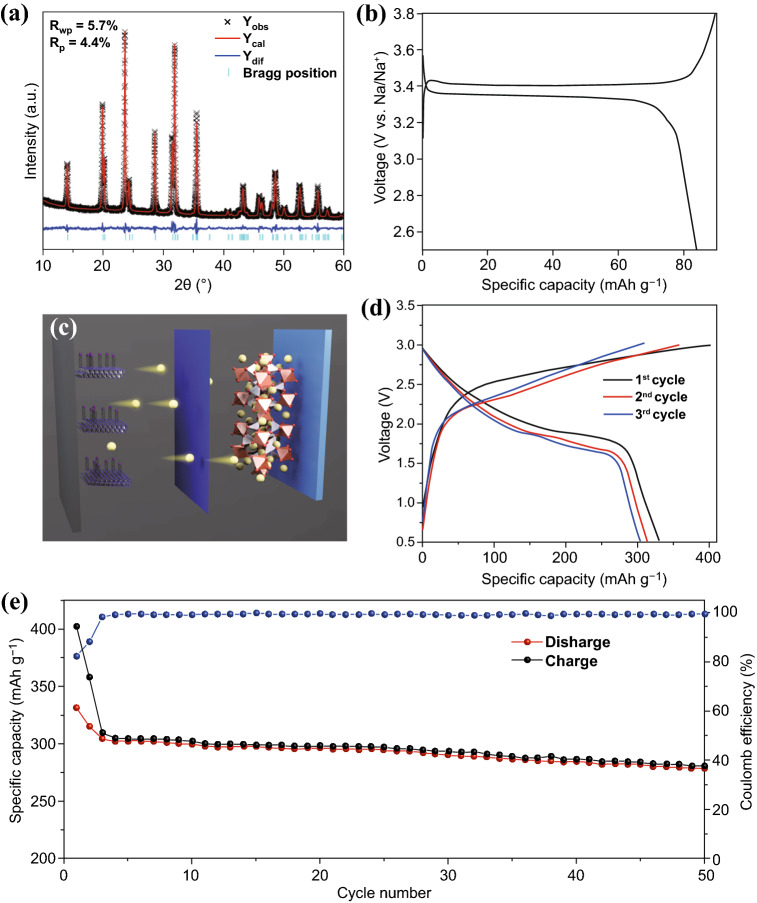
**a** XRD refinement of Na_3_V_2_ (PO_4_)_3_. **b** First discharge–charge curves of Na_3_V_2_ (PO_4_)_3_ half-cell at the current of 100 mA g^−1^. **c** Model structure of Na_3_V_2_ (PO_4_)//CoSe_2_@CNTs–MXene full cell. **d** Discharge–charge curves of Na_3_V_2_ (PO_4_)//CoSe_2_@CNTs–MXene full cell at the current of 100 mA g^−1^. **e** Cycle performance of Na_3_V_2_(PO_4_)//CoSe_2_@CNTs–MXene full cell at 100 mA g^−1^

To confirm the application value of CoSe_2_@CNTs–MXene as the anode in SIBs, Na_3_V_2_ (PO_4_)_3_/C cathode and CoSe_2_@CNTs–MXene anode full cell was assembled with NaPF_6_ in DEGDME as the electrolyte (Fig. 7c). The synthesis strategy of Na_3_V_2_ (PO_4_)_3_/C was reported in our previous work [47]. Figure 7a displays the experimental XRD pattern and calculated data of Na_3_V_2_ (PO_4_)_3_, with low *R*_wp_ (5.7%) and *R*_p_ (4.4%), indicating the high purity of Na_3_V_2_ (PO_4_)_3_ phase. Lattice parameters of Na_3_V_2_ (PO_4_)_3_ are *a* = 8.712798 Å, *b* = 8.712798 Å, *c* = 21.804346 Å with *α* = 90°, *β* = 90°, *λ* = 120° (R-3c), respectively, which can greatly match with preceding literature. Capacity–voltage curve of Na_3_V_2_ (PO_4_)_3_/C at the first cycle (Fig. 7b) shows the voltage plateau is at 3.4 V and Na_3_V_2_(PO_4_)_3_/C can provide an 80 mAh g^−1^ at 100 mA g^−1^ after 50 cycling periods (Fig. S18). For Na_3_V_2_ (PO_4_)_3_/C//CoSe_2_@CNTs–MXene full sodium-ion batteries, the cell can provide an initial charge/discharge capacity (Fig. 7d), 401 and 331 mAh g^−1^, under the current of 100 mA g^−1^, respectively, from 0.5 to 3 V (based on the weight of anode). Beyond that, the full cell can obtain an invertible capacity of about 280 mAh g^−1^ after 50 cycles (Fig. 7e), indicating the good application prospect in energy storage fields.

## Conclusions

Dimensional gradient structure of CoSe_2_@CNTs–MXene transferred from ZIF-67/MXene has been successfully designed. A particular “sheet–tube–dots” hierarchical structure can greatly promote the fast ion/electronic transmission and keep the stability of CoSe_2_ nanoparticles. Meanwhile, electrochemical performances of CoSe_2_@CNTs–MXene in two-electrode systems, ether/ester electrolyte systems were systematically explored. CoSe_2_@CNTs–MXene in ether exhibits outstanding cycling properties which can obtain 400 mAh g^−1^ after 200 cycles at 2 A g^−1^ with a high ICE of 81.7%, and excellent rate stability of 347.5 mAh g^−1^ at 5 A g^−1^ is much better than electrochemical behaviors in ester system (only 27 mAh g^−1^ for 200 cycles). Transformation mechanisms of CoSe_2_ were also explored by in situ XRD and ex situ Raman. Density functional theory study discloses that the CoSe_2_@CNTs–MXene in ether electrolyte system contributes to stable sodium storage performance owing to the strong adsorption force from hierarchical structure and weak interaction between electrolyte/electrode. For full cell, CoSe_2_@CNTs–MXene//Na_3_V_2_ (PO_4_)_3_/C can also afford a reversible capacity of 280 mAh g^−1^ over 50 cycles at a current of 100 mA g^−1^. Briefly, unique dimensional gradient structure and suitable electrolyte design promote potential application of CoSe_2_@CNTs–MXene in sodium storage fields.

## Electronic supplementary material

Below is the link to the electronic supplementary material.

Supplementary file 1 (PDF 1329 kb)

## References

[1] Huang Y, Zheng Y, Li X, Adams F, Luo W (2018). Electrode materials of sodium-ion batteries toward practical application. ACS Energy Lett..

[2] Schmuch R, Wagner R, Hörpel G, Placke T, Winter M (2018). Performance and cost of materials for lithium-based rechargeable automotive batteries. Nat. Energy.

[3] Vaalma C, Buchholz D, Weil M, Passerini S (2018). A cost and resource analysis of sodium-ion batteries. Nat. Rev. Mater..

[4] Xu G-L, Amine R, Abouimrane A, Che H, Dahbi M (2018). Challenges in developing electrodes, electrolytes and diagnostics tools to understand and advance sodium-ion batteries. Adv. Energy Mater..

[5] Li L, Zheng Y, Zhang S, Yang J, Shao Z (2018). Recent progress on sodium ion batteries: Potential high-performance anodes. Energy Environ. Sci..

[6] You Y, Manthiram A (2018). Progress in high-voltage cathode materials for rechargeable sodium-ion batteries. Adv. Energy Mater..

[7] Xu X, Zhou D, Qin X, Lin K, Kang F (2018). A room-temperature sodium–sulfur battery with high capacity and stable cycling performance. Nat. Commun..

[8] Xu X, Lin K, Zhou D, Liu Q, Qin X (2020). Quasi-solid-state dual-ion sodium metal batteries for low-cost energy storage. Chem.

[9] Ma J-L, Meng F-L, Yu Y, Liu D-P, Yan J-M (2019). Prevention of dendrite growth and volume expansion to give high-performance aprotic bimetallic Li-Na alloy–O_2_ batteries. Nat. Chem..

[10] Li Y, Lu Y, Zhao C, Hu Y-S, Titirici M-M (2017). Recent advances of electrode materials for low-cost sodium-ion batteries towards practical application for grid energy storage. Energy Storage Mater..

[11] Choudhary N, Islam MA, Kim JH, Ko T-J, Schropp A (2018). Two-dimensional transition metal dichalcogenide hybrid materials for energy applications. Nano Today.

[12] Kang W, Wang Y, Xu J (2017). Recent progress in layered metal dichalcogenide nanostructures as electrodes for high-performance sodium-ion batteries. J. Mater. Chem. A.

[13] Lv R, Robinson JA, Schaak RE, Sun D, Sun Y (2015). Transition metal dichalcogenides and beyond: Synthesis, properties and applications of single- and few-layer nanosheets. Acc. Chem. Res..

[14] Yang L, Hong W, Tian Y, Zou G, Hou H (2020). Heteroatom-doped carbon inlaid with Sb_2_X_3_ (X=S, Se) nanodots for high-performance potassium-ion batteries. Chem. Engin. J..

[15] Hu H, Zhang J, Guan B, Lou XW (2016). Unusual formation of CoSe@carbon nanoboxes, which have an inhomogeneous shell, for efficient lithium storage. Angew. Chem. Int. Ed..

[16] Zhong M, Kong L, Li N, Liu Y-Y, Zhu J (2019). Synthesis of MOF-derived nanostructures and their applications as anodes in lithium and sodium ion batteries. Coord. Chem. Rev..

[17] Wang H, Jiang Y, Manthiram A (2018). Long cycle life, low self-discharge sodium–selenium batteries with high selenium loading and suppressed polyselenide shuttling. Adv. Energy Mater..

[18] He Y, Wang L, Dong C, Li C, Ding X (2019). In-situ rooting ZnSe/n-doped hollow carbon architectures as high-rate and long-life anode materials for half/full sodium-ion and potassium-ion batteries. Energy Storage Mater..

[19] Yang J, Gao H, Men S, Shi Z, Lin Z (2018). CoSe_2_ nanoparticles encapsulated by *n*-doped carbon framework intertwined with carbon nanotubes: High-performance dual-role anode materials for both Li- and Na-ion batteries. Adv. Sci..

[20] Goodenough JB, Park K-S (2013). The Li-ion rechargeable battery: A perspective. J. Am. Chem. Soc..

[21] Xu K (2004). Nonaqueous liquid electrolytes for lithium-based rechargeable batteries. Chem. Rev..

[22] Zhang J, Wang D-W, Lv W, Qin L, Niu S (2018). Ethers illume sodium-based battery chemistry: Uniqueness, surprise and challenges. Adv. Energy Mater..

[23] Huang Y, Zhao L, Li L, Xie M, Wu F (2019). Electrolytes and electrolyte/electrode interfaces in sodium-ion batteries: From scientific research to practical application. Adv. Mater..

[24] Lin Z, Xia Q, Wang W, Li W, Chou S (2019). Recent research progresses in ether—and ester-based electrolytes for sodium-ion batteries. InfoMat.

[25] Su D, Kretschmer K, Wang G (2016). Improved electrochemical performance of Na-ion batteries in ether-based electrolytes: A case study of ZnS nanospheres. Adv. Energy Mater..

[26] Chen J, Mohrhusen L, Ali G, Li S, Chung KY (2019). Electrochemical mechanism investigation of Cu_2_MoS_4_ hollow nanospheres for fast and stable sodium ion storage. Adv. Funct. Mater..

[27] Giannozzi P, Andreussi O, Brumme T, Bunau O, Buongiorno Nardelli M (2017). Advanced capabilities for materials modelling with quantum espresso. J. Phys. Condes. Matter..

[28] Dion M, Rydberg H, Schröder E, Langreth DC, Lundqvist BI (2004). Van der Waals density functional for general geometries. Phys. Rev. Lett..

[29] Toby B (2001). EXPGUI, a graphical user interface for GSAS. J. Appl. Crystallogr..

[30] Alhabeb M, Maleski K, Anasori B, Lelyukh P, Clark L (2017). Guidelines for synthesis and processing of two-dimensional titanium carbide (Ti_3_C_2_Tx MXene). Chem. Mat..

[31] Ghidiu M, Lukatskaya MR, Zhao M-Q, Gogotsi Y, Barsoum MW (2014). Conductive two-dimensional titanium carbide ‘clay’ with high volumetric capacitance. Nature.

[32] Zhao X, Vashisth A, Prehn E, Sun W, Shah SA (2019). Antioxidants unlock shelf-stable Ti_3_C_2_Tx (MXene) nanosheet dispersions. Matter.

[33] Naguib M, Kurtoglu M, Presser V, Lu J, Niu J (2011). Two-dimensional nanocrystals produced by exfoliation of Ti_3_AlC_2_. Adv. Mater..

[34] Mashtalir O, Naguib M, Mochalin VN, Y. Dall’Agnese, M. Heon,  (2013). Intercalation and delamination of layered carbides and carbonitrides. Nat. Commun..

[35] Lv L-P, Zhi C, Gao Y, Yin X, Hu Y (2019). Hierarchical “tube-on-fiber” carbon/mixed-metal selenide nanostructures for high-performance hybrid supercapacitors. Nanoscale.

[36] Zhang W, Jiang X, Wang X, Kaneti YV, Chen Y (2017). Spontaneous weaving of graphitic carbon networks synthesized by pyrolysis of ZIF-67 crystals. Angew. Chem. Int. Ed..

[37] Meng J, Niu C, Xu L, Li J, Liu X (2017). General oriented formation of carbon nanotubes from metal–organic frameworks. J. Am. Chem. Soc..

[38] He Y, Luo M, Dong C, Ding X, Yin C (2019). Coral-like Ni_x_Co_1−x_Se_2_ for Na-ion battery with ultralong cycle life and ultrahigh rate capability. J. Mater. Chem. A.

[39] Gao J, Li Y, Shi L, Li J, Zhang G (2018). Rational design of hierarchical nanotubes through encapsulating Cose_2_ nanoparticles into MoSe_2_/C composite shells with enhanced lithium and sodium storage performance. ACS Appl. Mater. Interfaces.

[40] Huang H, Cui J, Liu G, Bi R, Zhang L (2019). Carbon-coated MoSe_2_/MXene hybrid nanosheets for superior potassium storage. ACS Nano.

[41] Park S-K, Kang YC (2018). MOF-templated n-doped carbon-coated CoSe_2_ nanorods supported on porous CNT microspheres with excellent sodium-ion storage and electrocatalytic properties. ACS Appl. Mater. Interfaces.

[42] Wang D, Li F, Lian R, Xu J, Kan D (2019). A general atomic surface modification strategy for improving anchoring and electrocatalysis behavior of Ti_3_C_2_T_2_ MXene in lithium–sulfur batteries. ACS Nano.

[43] Wang X, Yang C, Xiong X, Chen G, Huang M (2019). A robust sulfur host with dual lithium polysulfide immobilization mechanism for long cycle life and high capacity Li-S batteries. Energy Storage Mater..

[44] Fang G, Wang Q, Zhou J, Lei Y, Chen Z (2019). Metal organic framework-templated synthesis of bimetallic selenides with rich phase boundaries for sodium-ion storage and oxygen evolution reaction. ACS Nano.

[45] Hou H, Banks CE, Jing M, Zhang Y, Ji X (2015). Carbon quantum dots and their derivative 3d porous carbon frameworks for sodium-ion batteries with ultralong cycle life. Adv. Mater..

[46] Taberna PL, Simon P, Fauvarque JF (2003). Electrochemical characteristics and impedance spectroscopy studies of carbon-carbon supercapacitors. J. Electrochem. Soc..

[47] Wang H, Jiang D, Zhang Y, Li G, Lan X (2015). Self-combustion synthesis of Na_3_V_2_(PO_4_)_3_ nanoparticles coated with carbon shell as cathode materials for sodium-ion batteries. Electrochim. Acta.

