# Supplementary material for: Dimensional Gradient Structure of CoSe2@CNTs–MXene Anode Assisted by Ether for High-Capacity, Stable Sodium Storage
Source: Nanomicro Lett. 2021 Jan 4;13:40. doi: 10.1007/s40820-020-00562-7 (PMC8187670; doi:10.1007/s40820-020-00562-7)
Supplement: Supplementary file 1 — (PDF 1329 kb) [file 40820_2020_562_MOESM1_ESM.pdf]

Supporting Information for

Dimensional Gradient Structure of $CoSe_2$@CNTs-MXene Anode Assisted by Ether for High Capacity, Stable Sodium Storage

Enze Xu[1], Pengcheng Li[1], Junjie Quan[1], Hanwen Zhu[1], Li Wang[2], Yajing Chang[3], Zhenjie Sun[1], Lei Chen[1], Dabin Yu[3,*], Yang Jiang[1,*]

[1]School of Materials Science and Engineering, Hefei University of Technology, Hefei 230009, P. R. China

[2]School of Chemistry and Chemical Engineering, Hefei University of Technology, Hefei 230009, P. R. China

[3]State Key Laboratory of Pulsed Power Laser Technology, National University of Defense Technology, Hefei 230037, P. R. China

*Corresponding authors. E-mail: apjiang@hfut.edu.cn (Yang Jiang); dabinyu@sina.cn (Dabin Yu)

S1 Supplementary Tables and Figures

Table S1 Impedance parameters for the equivalent circuits

	Inductor (nH)	R_1 (Ω)	R_2 (Ω)	CPE_1		CPE_2	
				V(mF)	exponent(m)	V(μF)	exponent(m)
$CoSe_2$@CNTs-MXene (ether)	441	8.55	9.77	313	898	688	606
$CoSe_2$@CNTs-MXene (ester)	421	17.5	239	280	512	14.7	807
$CoSe_2$@CNTs	288	14.2	11.7	2.61	799	207	763

Table S2 Comparison of MXene-based anode in sodium-ion storage

**Materials**	**Performance**		**References**
	**Cycling***	**Rate****	
$CoSe_2$@CNTs-MXene	400/2/200th	347.5/5	This work
Phosphorene/ $Ti_3C_2T_x$	343/1/1000th	193/5	[S1]
3D carbon coated MXene	337.9/0.64/600th	194.7/3.2	[S2]
CT-S@ Ti_3C_2-450	492/0.1/100th	223/5	[S3]
Hollow MXene Spheres	210/0.5/1000th	120/5	[S4]
$NaTi_2(PO_4)_3$ cubes on Ti_3C_2	150/1/2000th	113/5	[S5]
MXene-Hard Carbon	267.9/0.2/1500th	98.2/2	[S6]
Ti_3C_2-NiCoP	302.8/0.1/100th	240.1/2	[S7]
Ti_3C_2 MXene-Derived Sodium Titanate Nanoribbons	191/0.2/150th	101/2	[S8]

*): Capacity (mAh g^{-1})/Current Density (A g^{-1})/Cycles; **): Capacity (mAh g^{-1})/Current Density (A g^{-1});

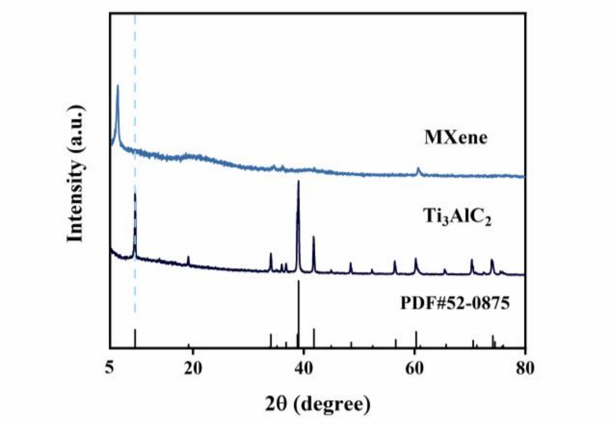

Fig. S1 XRD patterns of Ti_3AlC_2 and MXene

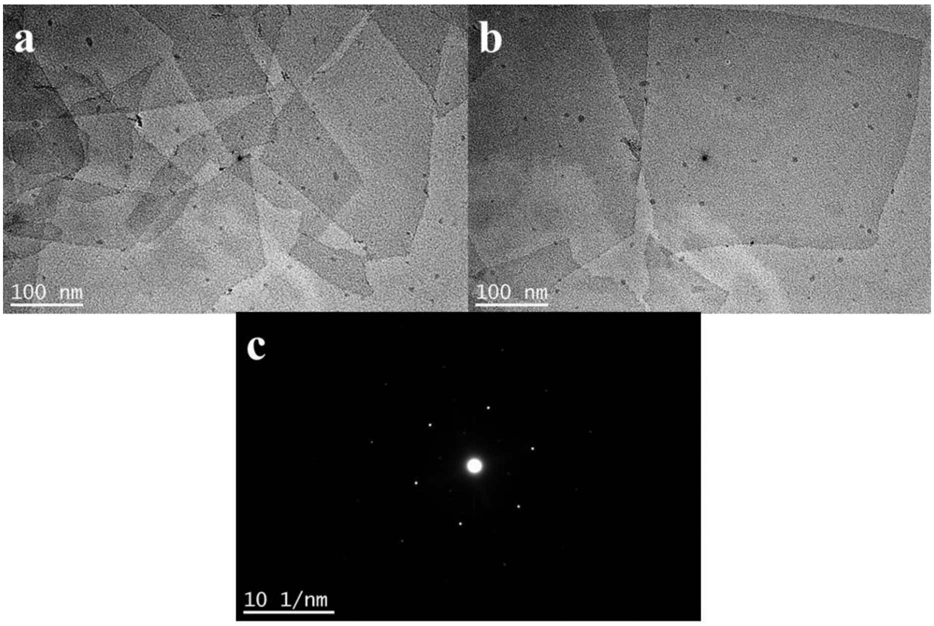

Fig. S2 a, b TEM images of $Ti_3C_2T_x$ MXene nanosheets. **c** SEAD patterns of single-layer $Ti_3C_2T_x$ MXene nanosheet

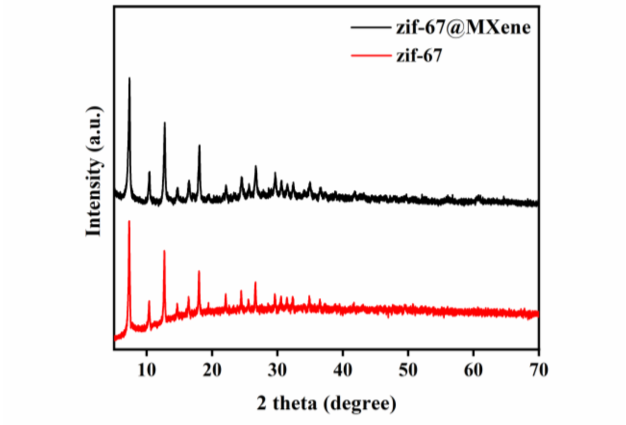

Fig. S3 XRD patterns of ZIF-67 and ZIF-67/MXene

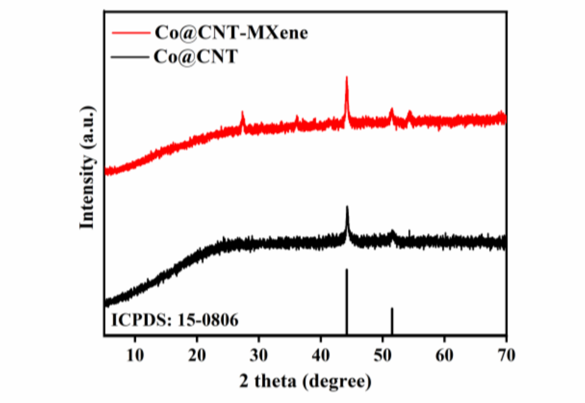

Fig. S4 XRD patterns of Co@CNTs and Co@CNTs-MXene after annealing treatment at 800 °C under Ar/H_2 atmosphere

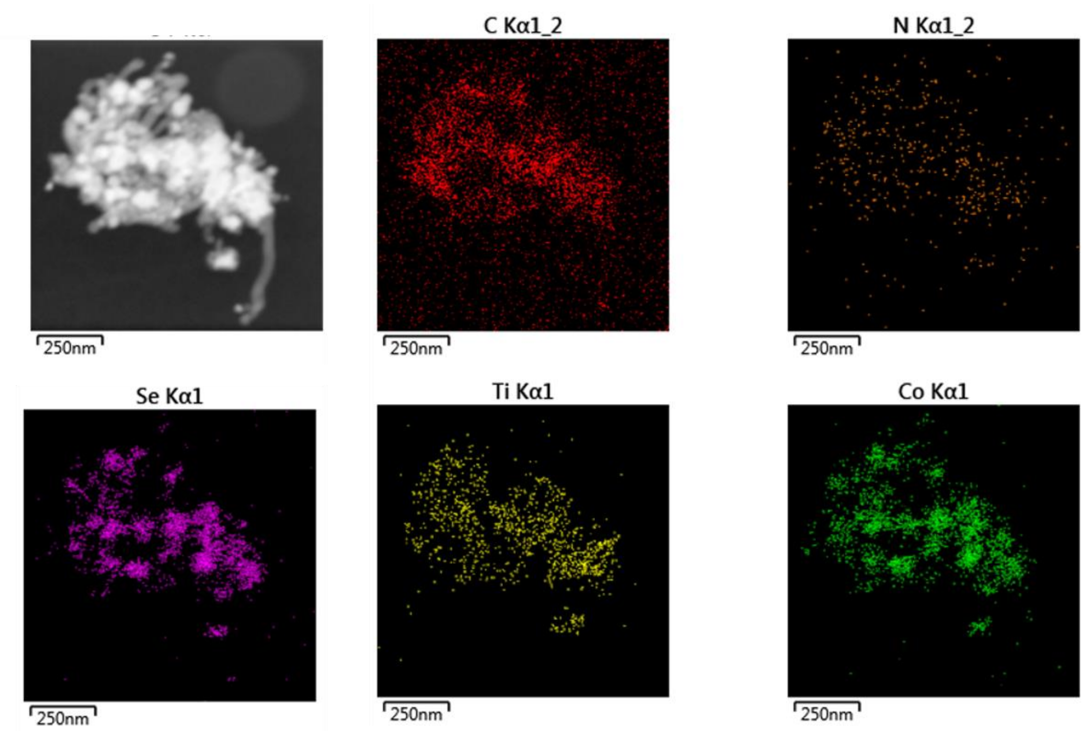

Fig. S5 Elemental Mapping of $CoSe_2$@CNTs-MXene

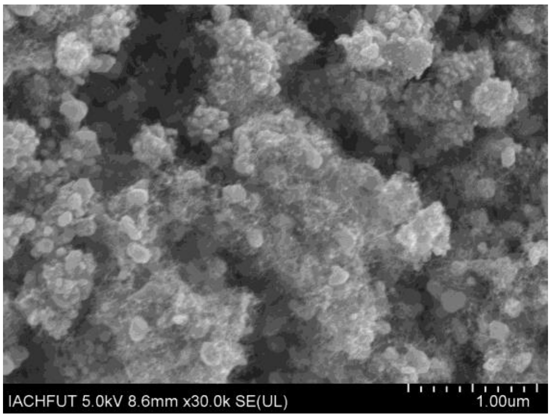

Fig. S6 SEM images of $CoSe_2$@CNTs

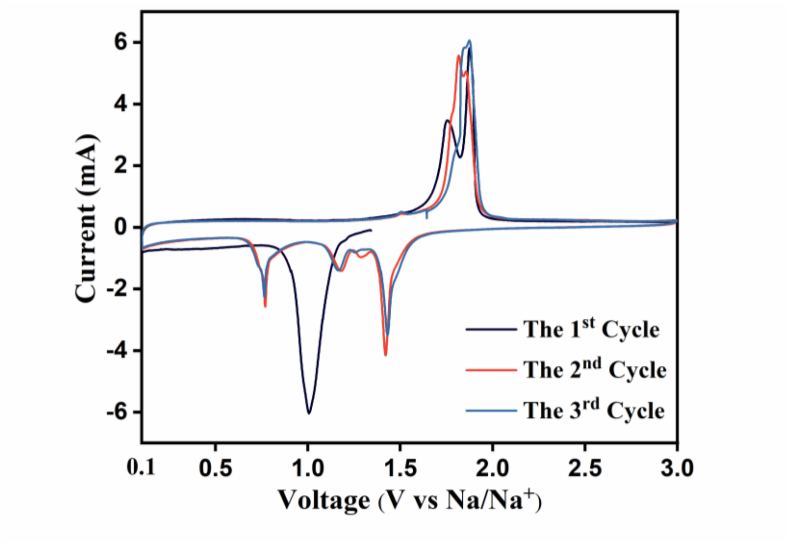

Fig. S7 CV curves of $CoSe_2$@CNTs

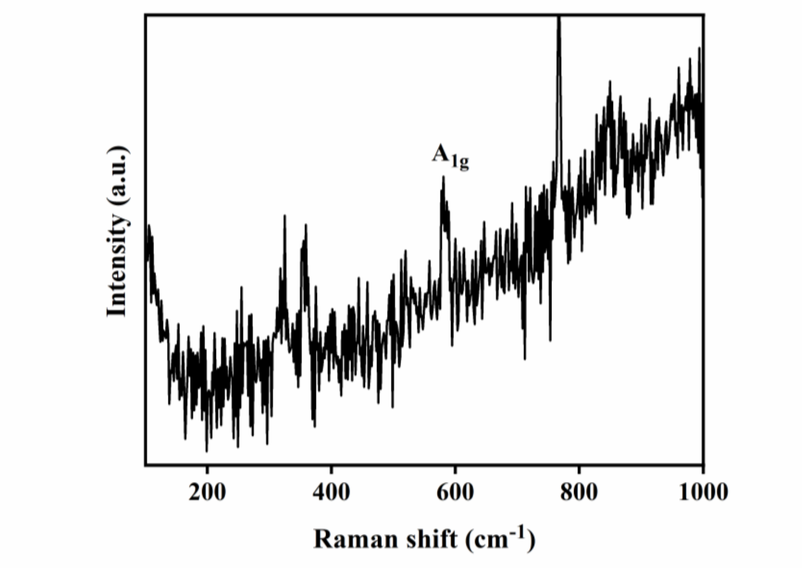

Fig. S8 Raman of $CoSe_2$ after charging to 3.0 V

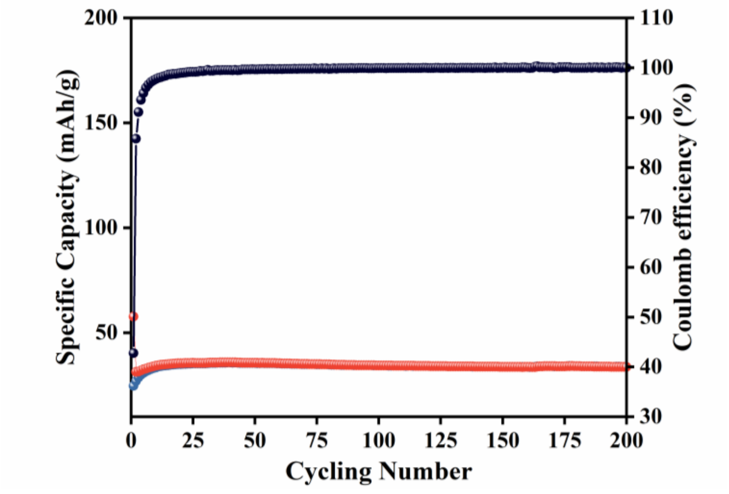

Fig. S9 Cycle performance of pure MXene

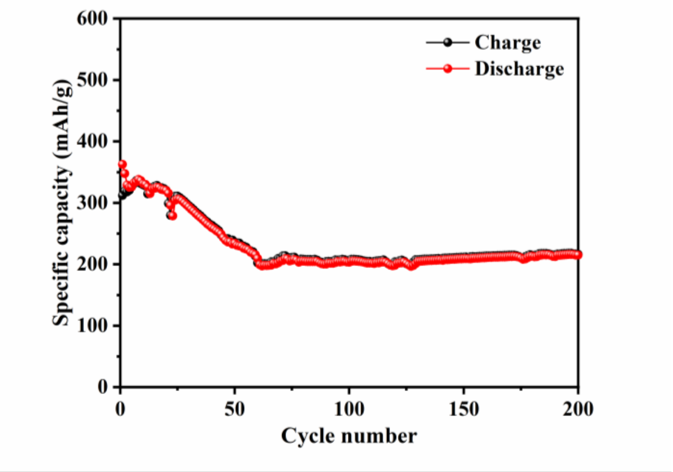

Fig. S10 Cycle performance of $CoSe_2$@CNTs

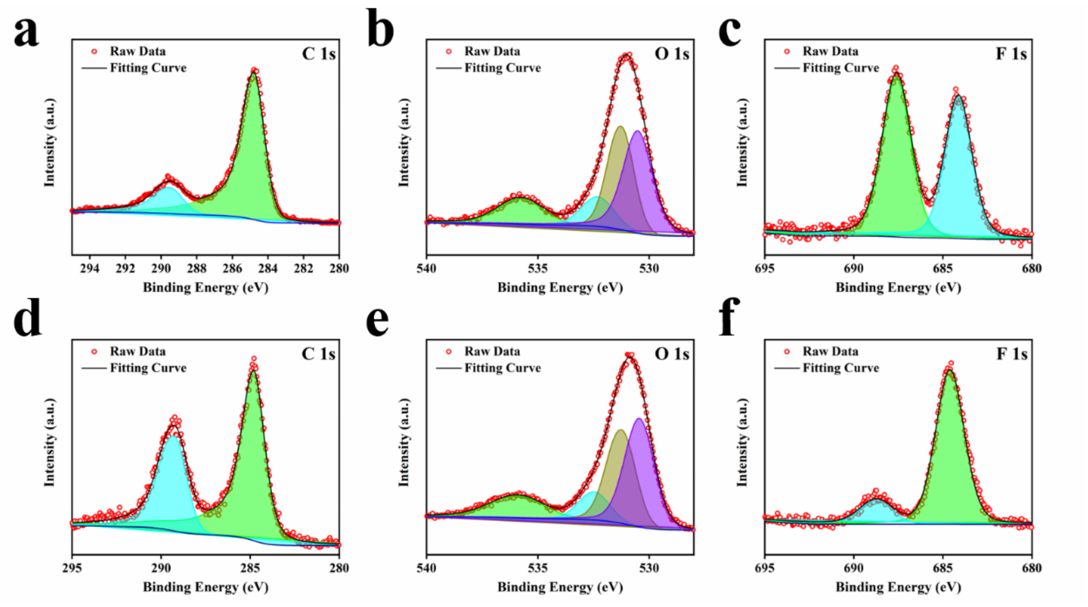

Fig. S11 XPS spectrum **a, d**) C 1s, **b, e**) O 1s, **c, f**) F 1s of electrode surface with ether and ester electrolyte systems

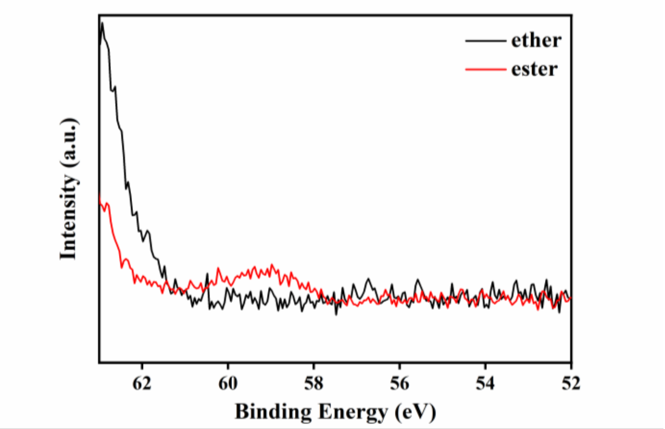

Fig. S12 XPS spectrum of separators with ether and ester electrolyte systems

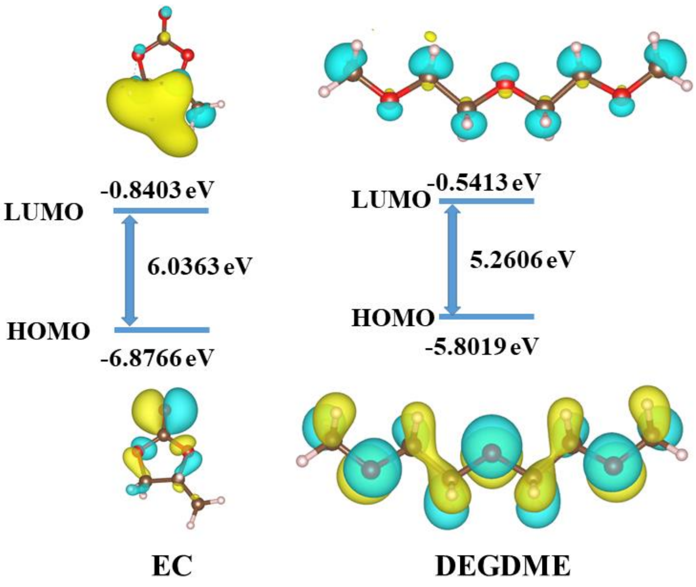

Fig. S13 Diagram of LUMO and HOMO energy level of Propylene carbonate (PC) and Bis(2-methoxy ethyl)ether (DEGDME)

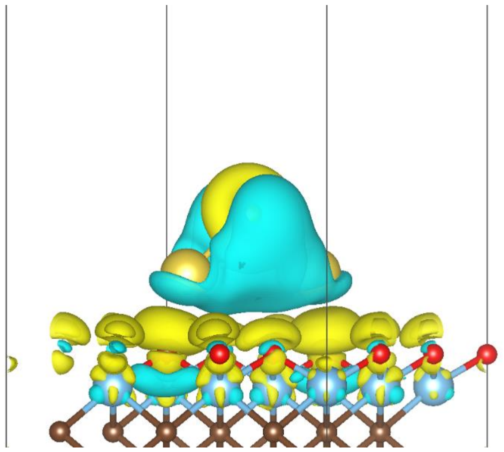

Fig. S14 Charge density difference of Na_2Se on $Ti_3C_2O_2$

S2 Calculation Method

Capacitive contribution can be calculated by the following equation:

$$i=av^{b} \quad \text{(S1)}$$

Where i is the current (A),

v is the scan rate (mV/s).

The slope b is 0.5 demonstrates a diffusion-controlled process (battery-type behavior). When slope is 1, this means a non-diffusion-controlled redox reactions on the surface (capacitive effect).

$$i=k_1v+k_2v^{1/2} \quad \text{(S2)}$$

In Eq. S2, k_1v and $k_2v^{1/2}$ correspond to the current contribution from the capacitive effect and diffusion-controlled process, respectively.

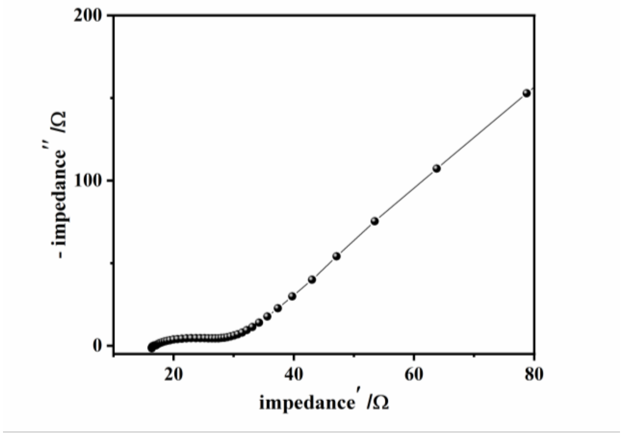

Fig. S15 Nyquist plots of $CoSe_2$@CNTs

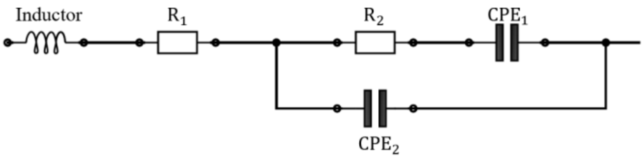

Fig. S16 Equivalent circuit of EIS

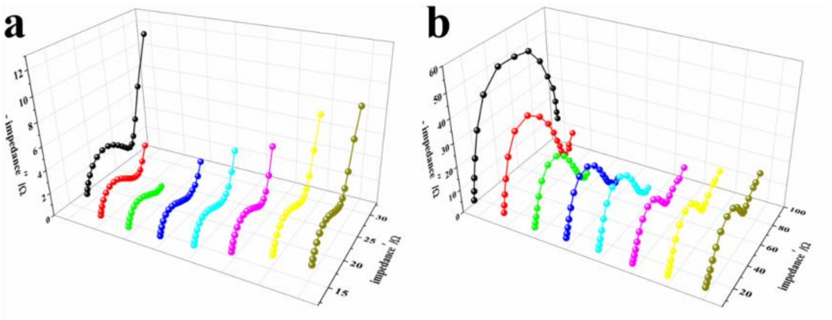

Fig. S17 Dynamic EIS analysis of $CoSe_2$@CNTs-MXene in ether and ester electrolyte at first discharge/charge cycle

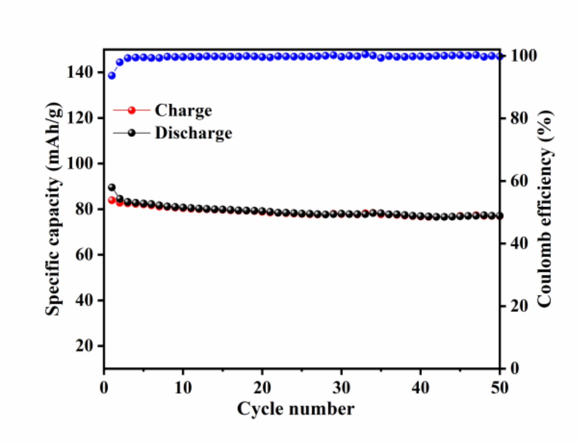

Fig. S18 Cycle performance of $Na_3V_2(PO_4)_3$ half cell at the current of 100 mAh g^{-1}

Supplementary References

[S1]X. Guo, W. Zhang, J. Zhang, D. Zhou, X. Tang et al., Boosting sodium storage in two-dimensional phosphorene/$Ti_3C_2T_x$ mxene nanoarchitectures with stable fluorinated interphase. ACS Nano **14**(3), 3651-3659 (2020). https://doi.org/10.1021/acsnano.0c00177

[S2]P. Zhang, R.A. Soomro, Z. Guan, N. Sun, B. Xu, 3d carbon-coated mxene architectures with high and ultrafast lithium/sodium-ion storage. Energy Storage Mater. **29**, 163-171 (2020). https://doi.org/10.1016/j.ensm.2020.04.016

[S3]J. Luo, J. Zheng, J. Nai, C. Jin, H. Yuan et al., Atomic sulfur covalently engineered interlayers of Ti_3C_2 mxene for ultra-fast sodium-ion storage by enhanced pseudocapacitance. Adv. Funct. Mater. **29**(10), 1808107 (2019). https://doi.org/10.1002/adfm.201808107

[S4]M.-Q. Zhao, X. Xie, C.E. Ren, T. Makaryan, B. Anasori et al., Hollow MXene spheres and 3d macroporous mxene frameworks for na-ion storage. Adv. Mater. **29**(37), 1702410 (2017). https://doi.org/10.1002/adma.201702410

[S5]Q. Yang, T. Jiao, M. Li, Y. Li, L. Ma et al., In situ formation of $NaTi_2(PO_4)_3$ cubes on Ti_3C_2 mxene for dual-mode sodium storage. J. Mater. Chem. A **6**(38), 18525-18532 (2018). https://doi.org/10.1039/C8TA06995F

[S6]N. Sun, Q. Zhu, B. Anasori, P. Zhang, H. Liu et al., Mxene-bonded flexible hard carbon film as anode for stable na/k-ion storage. Adv. Funct. Mater. **29**(51), 1906282 (2019). https://doi.org/10.1002/adfm.201906282

[S7]D. Zhao, R. Zhao, S. Dong, X. Miao, Z. Zhang et al., Alkali-induced 3d crinkled porous Ti_3C_2 mxene architectures coupled with nicop bimetallic phosphide nanoparticles as anodes for high-performance sodium-ion batteries. Energy Environ. Sci. **12**(8), 2422-2432 (2019). https://doi.org/10.1039/C9EE00308H

[S8]P. Lian, Y. Dong, Z.-S. Wu, S. Zheng, X. Wang et al., Alkalized Ti_3C_2 mxene nanoribbons with expanded interlayer spacing for high-capacity sodium and potassium ion batteries. Nano Energy **40**, 1-8 (2017). https://doi.org/10.1016/j.nanoen.2017.08.002
